# Drug Physicochemical Properties and Capsule Fill Determine Extent of Premature Gastric Release from Enteric Capsules

**DOI:** 10.3390/pharmaceutics14112505

**Published:** 2022-11-18

**Authors:** Fouad S. Moghrabi, Hala M. Fadda

**Affiliations:** Department of Pharmaceutical Sciences, College of Pharmacy and Health Sciences, Butler University, Indianapolis, IN 46208, USA

**Keywords:** delayed release, modified release, gastroresistant, pH dependent, peptide, hypochlorhydria

## Abstract

Intrinsically, enteric capsule shells offer several advantages compared to coating of dosage forms with enteric polymers. We undertook a systematic investigation to elucidate capsule-fill parameters that may result in premature gastric drug release from Vcaps^®^ Enteric capsules (Lonza CHI, Morristown, NJ, USA). Four model drugs with different ionization and solubility profiles were investigated: acetaminophen, ketoprofen, trimethoprim and atenolol. Different fill loads, diluents and drug-to-diluent ratios were explored. Enteric capsules were filled with drug or drug and diluent powder mix and underwent USP II dissolution testing using mini-vessels and paddles. Capsules were tested in pH 2 (0.01 M HCl) or pH 4.5 (3.2 × 10^−5^ M HCl) 200 mL acid media to simulate normal, fasted or hypochlorhydric gastric pH, respectively. Acetaminophen, trimethoprim and atenolol displayed premature gastric drug release from enteric capsules. The extent of premature release was dependent on drug solubility, ionization profile and capsule-fill level. At 100 mg drug-fill level, acetaminophen, trimethoprim and atenolol gave rise to 10.6, 12.2 and 83.1% drug release, respectively, in normal, fasted, gastric fluids. Diffusion layer pH of trimethoprim and atenolol in pH 2 media was determined to be pH 6.3 and 10.3, respectively. Upon increasing capsule-fill load using microcrystalline cellulose as a diluent, a significant reduction in premature gastric release was observed. However, including mannitol as a diluent was only effective at decreasing premature drug release at a low drug-to-diluent ratio. Systematic in vitro screening of enteric capsule fills needs to be conducted to ensure that drug product performance is not compromised.

## 1. Introduction

Gastroresistant or enteric dosage forms provide effective delivery systems for acid/enzyme labile drugs, including small molecules, peptides and monoclonal antibodies. They can also serve to protect the stomach from adverse effects associated with some drugs, such as aspirin, non-steroidal anti-inflammatory drugs (NSAIDs) and erythromycin. Furthermore, enteric dosage forms enable direct drug targeting to the small intestine or colon, important for delivery of steroids and mesalamine (5-aminosalicylic acid) to treat inflammatory bowel diseases. The potential of enteric formulations to improve the absorption and reduce variability in weak acid drugs by enabling supersaturation in the small intestine has also been demonstrated [1].

Typically, gastroresistant dosage forms are achieved by coating tablets, pellets or capsules with enteric polymers. Optimization of the coating process needs to be carefully controlled to achieve uniform film coating thickness and optimal drug release. Capsule shells that are intrinsically enteric can offer several advantages compared to coating of dosage forms with enteric polymers. Enteric capsule shells allow for rapid prototype development, avoid the need for process development and scale up of the coating process and avoid exposure of drug product to heat during the coating process [2].

Enteric capsules using a combination of polymers have been developed via the dip-coating method. These include capsules produced from a combination of hydroxypropyl methylcellulose acetate succinate (HPMC AS), hydroxypropyl methylcellulose (HMPC) and methacrylic acid copolymers Eudragit L100 and S100 [3]. Capsules comprising Eudragit L100-55 and zein, a corn protein with a high content of hydrophobic amino acids, have also been examined in vitro [4]. Enteric capsules have also been fabricated using fused-deposition 3D printing with Eudragit^®^ L100-55, polyethylene glycol 400 as a plasticizer and polylactic acid as a thermoplastic polymer [5].

To date, several enteric, ready-to-fill capsules are commercially available, which claim to prevent gastric drug release. These include: Bio-VXR^®^ (BioCaps, El Monte, CA, USA) with an undisclosed formulation of vegetable capsules, DRcap^TM^ (Lonza Capsules and Health Ingredients, Morristown, NJ, USA) nutraceutical capsules composed of HPMC and gellan, designed to swell and delay disintegration, enTrinsic^TM^ drug delivery capsules (Lonza) composed of cellulose acetate phthalate (CAP) and Vcaps^®^ Enteric capsules (Lonza) composed of HPMC, HPMC-AS polymers and gellan gum as the gelling agent [6,7]. In 2021, EUDRACAP^TM^ (Evonik, Darmstadt, Germany) HPMC capsules coated with methacrylic acid copolymers that can easily be opened and closed were launched [8,9].

The United States Pharmacopeia (USP) guidelines determine that drug release from enteric preparations should be <10% upon exposure to the acid phase in a USP II dissolution test. Failure to provide acid protection has been reported for enteric capsules and we, therefore, undertook a systematic in vitro investigation to elucidate capsule-fill parameters that can result in premature gastric drug release from Vcaps Enteric capsules. Four model drugs with different ionization and aqueous solubility profiles were investigated, including: acetaminophen (practically non-ionizable at physiological pH range), trimethoprim and atenolol (weak bases with six-fold differences in solubility in acid media) and ketoprofen (weak acid). While diluents are known to affect drug release from immediate release tablets/capsules, the impact of diluents on premature gastric drug release from enteric capsules has not been previously reported and was, therefore, explored in our current study. Different capsule-fill levels, diluents and drug-to-diluent ratios were investigated. Model diluents selected were mannitol and microcrystalline cellulose, with good solubility and practical insolubility in water, respectively.

The effect of gastric media pH, specifically hypochlorhydric, simulated gastric fluids, on premature gastric drug release from enteric capsules was also examined. Hypochlorhydria is reduced gastric acid secretions, which leads to elevated gastric pH. The exploration of the impact of hypochlorhydria on drug release is pertinent, since the use of acid-reducing agents (ARAs), including proton pump inhibitors (PPIs) and H_2_-receptor antagonists, is prevalent in adult populations. For example, 75 million prescriptions for the PPI omeprazole were dispensed in the United States in 2014 [10]. Fasted pH in individuals on PPIs is typically in the range of pH 4–5 [11]. Furthermore, 10% of healthy adults over the age of 65 years in North America have a fasting gastric pH > 6 [12].

## 2. Materials and Methods

### 2.1. Materials

Vcaps^®^ Enteric capsules, size 0, were obtained from Lonza Capsules and Health Ingredients (Morristown, NJ, USA). Trimethoprim (≥98% purity) and ketoprofen (≥98% purity) were purchased from Cayman Chemical Company (Ann Arbor, MI, USA). Acetaminophen (98–102% pure powder) and atenolol (≥98% purity) were purchased from Sigma-Aldrich (St. Louis, MO, USA). Microcrystalline cellulose (MCC) (MICROCEL^®^ MC-101) and mannitol (PEARLITOL^®^ 100 SD) were received as a gift from Roquette (Keokuk, IA, USA). Sodium dihydrogen phosphate monohydrate, sodium hydroxide and 5 M HCL were purchased from VWR (Radnor, PA, USA). Deionized water was used throughout.

Dissolution accessories were purchased from Quality Lab Accessories (Telford, PA, USA), including: 200 mL vessels (175 mm height, 45 mm internal diameter) and mini-paddles (300 mm length, 9 mm height of paddle blade), USP compliant Japanese Pharmacopoeia sinker baskets and ultra-high-molecular-weight polyethylene (UHMPE) 10 μm porous filters.

### 2.2. Model Drugs

Four model drugs with different ionization and solubility profiles were investigated (Table 1). Acetaminophen is a BCS class III compound with antipyretic and analgesic properties. It is practically non-ionizable over the physiological pH range and, therefore, does not exhibit pH-dependent solubility [13]. It has a water solubility of 14 mg/mL [14]. Trimethoprim is a BCS class II antibiotic [15]. It is a weak base with a pKa of 7.12 [16] and displays a pH-dependent solubility profile. Solubility at pH 2 was experimentally determined in house to be 3.2 ± 0.04 mg/mL (experimental details provided in solubility assays section). Atenolol is a BCS class III beta-blocker with antiarrhythmic and antihypertensive properties [17]. It is a weak base with a pKa of 9.6 [16] and solubility of 20.4 ± 0.1 mg/mL in pH 2 media (experimentally determined in house). Ketoprofen is a BCS class II NSAID. It is a weak acid with a pKa of 4.39 and solubility of 0.21 mg/mL in pH 2 media [18] and 40.8 mg/mL in pH 6.8 media [19]. Dose/solubility ratios for 100 mg drug load at pH 2 are included in Table 1.

### 2.3. Methods

#### 2.3.1. Capsule-Fill Level and Diluents

To explore the effect of capsule-fill level on premature gastric drug release from enteric capsules, Vcaps Enteric capsules were manually filled with different drug loads of 450, 100 or 20 mg and underwent dissolution testing. In another set of experiments, the effect of diluents with different aqueous solubility on drug dissolution behavior from enteric capsules was also investigated. Among the tested drugs, atenolol and acetaminophen were mixed with MCC or mannitol. The diluents were geometrically mixed with the drug and included at different ratios to achieve a total fill weight of 300 mg. Fill compositions explored included 7% drug (20 mg) and 93% diluent (280 mg) or 33% drug (100 mg) and 67% diluent (200 mg).

#### 2.3.2. Dissolution Tests

Vcaps Enteric capsules underwent dissolution testing in USP II dissolution apparatus (SR8 PLUS USP II, Hanson Research Corporation, Chatsworth, CA, USA). Mini-vessel set-up was utilized to more closely simulate fasted stomach volumes. Mini-paddles were also utilized at a rotation speed of 50 rotations per minute. The paddles were calibrated and 10 μm porous filters were attached to the cannulas for in-line filtration. Capsules were placed in USP-compliant Japanese Pharmacopoeia Sinker Baskets and exposed to two hours of 200 mL of acid phase followed by 200 mL pH 6.8, 0.05 M phosphate buffer for an additional two hours. Acid phase comprised pH 2, 0.01 M HCl to simulate normal, fasted gastric pH or pH 4.5, 3.2 × 10^−5^ M HCl to simulate hypochlorhydric gastric pH. Temperature in the vessels was maintained at 37 °C. At the end of the acid stage of the dissolution test, the media were discarded and vessels were filled with 200 mL of pre-warmed buffer. All dissolution media were degassed and filtered using vacuum filtration system.

Sample volumes of 1 mL were taken every 30 min until the end of the test (4 h) and all samples were replaced with fresh, relevant media. Samples were filtered with 0.45 µm PVDF filters and the first 0.5 mL of filtrate was discarded and dilution of samples was carried out when necessary. Filter validation was carried out for all drugs to ensure no significant drug absorption takes place. Drug analysis was undertaken using UV-spectrophotometry (NanoDrop 2000 UV-Vis, Thermo Scientific, MA, USA). Acetaminophen was analyzed at 243 nm, trimethoprim at 270 nm, atenolol at 225 nm and ketoprofen at 258 nm. All experiments were carried out at least in triplicate and drug release expressed at a given time as dissolved mean % ± standard deviation (SD).

#### 2.3.3. Solubility Assays

Where drug solubility in gastric media was not reported in the literature, it was determined in house using the shake-flask method. Solubility of atenolol and trimethoprim in gastric phase media (pH 2 and pH 4.5) was determined by adding excess drug to the media and shaking for 12 h. Samples were taken at 2, 4, 6 and 12 h until equilibrium solubility was achieved. Samples were filtered with 0.45 µm PVDF filters, the first 0.5 mL of filtrate was discarded and the filtrate was diluted appropriately and analyzed using UV spectrophotometry. All solubility experiments were performed in triplicate.

#### 2.3.4. pH of Capsule Content on Exposure to Dissolution Media

To understand the pH changes within the capsule fill upon ingress of dissolution medium, 1 mL of pH 2 acid phase media was added to 25 mg of solid (drug alone or in the presence of diluent) and mixed with a vortex. The pH of the medium in contact with excess solid was measured using a calibrated pH meter. This measurement also corresponds to the micro-environmental pH or diffusion layer pH (pH*_d_*), since the diffusion layer of dissolving drug particles is saturated with dissolving solids [20,21,22].

## 3. Results

### 3.1. Effect of Drug Properties on Premature Drug Release in pH 2 Acid Phase

The extent of premature gastric drug release from Vcaps Enteric capsules was observed to be influenced by drug solubility and ionization profile. Drug ionization was found to impact pH*_d_*, as illustrated in Table 2. Since acetaminophen is non-ionizable, measured pH*_d_* was pH 2.3 ± 0.02, close to the pH of the gastric media. Trimethoprim pH*_d_* was measured to be 6.3 ± 0.07, which can be explained by the weak basic nature of the drug. Atenolol pH*_d_* was observed to be even higher at 10.3 ± 0.05, which may be attributed to the drug’s weak basic nature as well as high solubility in gastric media.

**Figure 1:**
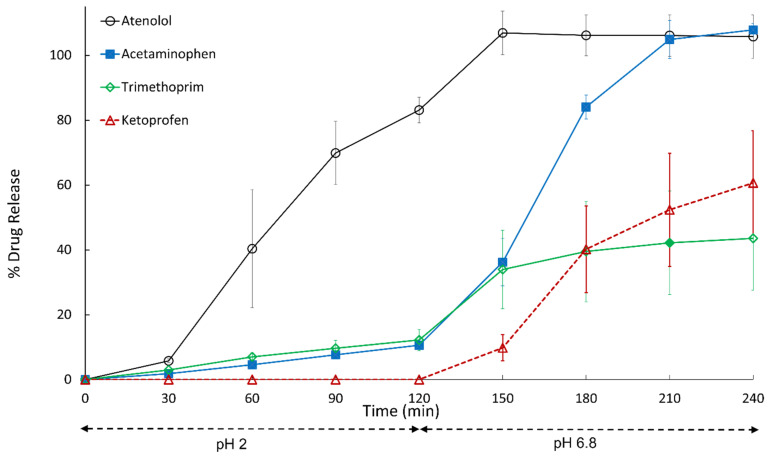
Dissolution profiles of Vcaps^®^ Enteric capsules filled with 100 mg of drug. Data shown as drug release mean % ± SD.

**Figure 2:**
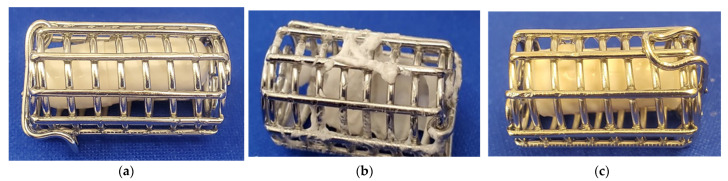
Vcaps^®^ Enteric capsules filled with 450 mg of drug after two hours of exposure to pH 2 acid phase: (**a**) Capsules filled with acetaminophen; (**b**) capsules filled with atenolol; (**c**) capsules filled with trimethoprim.

Acetaminophen, trimethoprim and atenolol displayed premature drug release from Vcaps Enteric capsules in the gastric phase of the dissolution test, while ketoprofen demonstrated no premature gastric release (Figure 1). The observed changes in pH at the end of the acid phase of the dissolution tests were measured to be <0.5 units for all the drugs tested. At the intermediate 100 mg capsule-fill level, acetaminophen and trimethoprim gave rise to 10.6 ± 0.9% and 12.2 ± 2.9% drug release, respectively, in acidic media, while atenolol displayed the greatest extent of premature drug release of 83.1 ± 4%. Gastric drug release from enteric capsules may be explained by hydration of capsule shell on exposure to aqueous media, thus, allowing for the ingress of aqueous fluid into capsule fill. Furthermore, the cap and body of the capsule deform at different rates (Figure 2a) and diffusion of the drug solution may take place through the junction at which they meet.

Acetaminophen displays good aqueous solubility and drug release may arise via diffusion of solubilized drug out of hydrated polymer capsule shell and at body/cap junction. A similar extent of premature gastric release is observed for trimethoprim and acetaminophen from enteric capsules at 100 mg and 20 mg drug loads. At 20 mg drug load, premature release was 42.5 ± 9.5% and 35.9 ± 8.3% for trimethoprim and acetaminophen, respectively (*p* > 0.05). This similar extent of premature drug release in acidic media may be explained by the higher solubility of acetaminophen (14 mg/mL) compared to trimethoprim (3.2 mg/mL) in the gastric phase, counteracting the higher pH*_d_*, 6.3 ± 0.07, of trimethoprim. At high capsule-fill levels of 450 mg, the extent of premature gastric release is 3.9 ± 1.2 and 4 ± 0.4% for acetaminophen and trimethoprim, respectively.

For all the tested atenolol capsule-fill levels (low, mid and high), premature gastric drug release and dissolution of the shell of Vcaps enteric in the acid phase were observed. The extent of premature drug release detected for the low, mid and high capsule fills was 86.3 ± 19.7, 83.1 ± 4 and 76.3 ± 9.7%, respectively. The higher extent of drug release from enteric capsules filled with atenolol may be explained by the drug’s weak basic nature and high solubility in acidic media (20.4 mg/mL), causing the pH*_d_* to reach 10.3 ± 0.05. pH*_d,_* therefore, exceeding the dissolution pH threshold of the enteric HPMC-AS polymer comprising the capsule shell. pH threshold of HPMC-AS ranges from 5.5 to 6.8, depending on polymer grade and, therefore, premature dissolution of capsule shell in the gastric phase compromises capsule integrity (Figure 2b). Capsule deformation may explain the high variability in atenolol release profiles observed during dissolution in the acid phase. Deformation of trimethoprim Vcaps Enteric capsules are also observed after 2 h of exposure to 0.01 M HCl, although to a lesser extent (Figure 2c).

The calculated dose-to-solubility ratios in Table 1 indicate the volume of media required to dissolve a given drug dose. At the 100 mg drug load, acetaminophen, trimethoprim and atenolol meet the Biopharmaceutics Classification System (BCS) criteria for high solubility (dose/solubility ratio < 250 mL) in pH 2 acidic media. The high solubility criteria are also met for the 20 and 450 mg drug loads at pH 2. Per the United States Pharmacopeia, sink conditions are achieved when the volume of dissolution media is at least three-times the volume required to form a saturated solution. Sink conditions were achieved for acetaminophen, trimethoprim and atenolol at 100 mg drug load in pH 2 acidic media; however, they were not achieved for ketoprofen. While high solubility and sink conditions criteria were not met for ketoprofen, this does not affect the conclusions, since no drug release was observed from ketoprofen enteric capsules at any of the drug loads in the acid phase of the dissolution test. The dissolution rate is fastest under sink conditions and, per the Noyes Whitney equation, as drug concentration (C_t_) rises, dissolution rate slows down and approaches zero as drug concentration reaches drug solubility (C_s_) [20].

### 3.2. Effect of Diluent on Premature Gastric Drug Release in pH 2 Acid Phase

**Figure 3:**
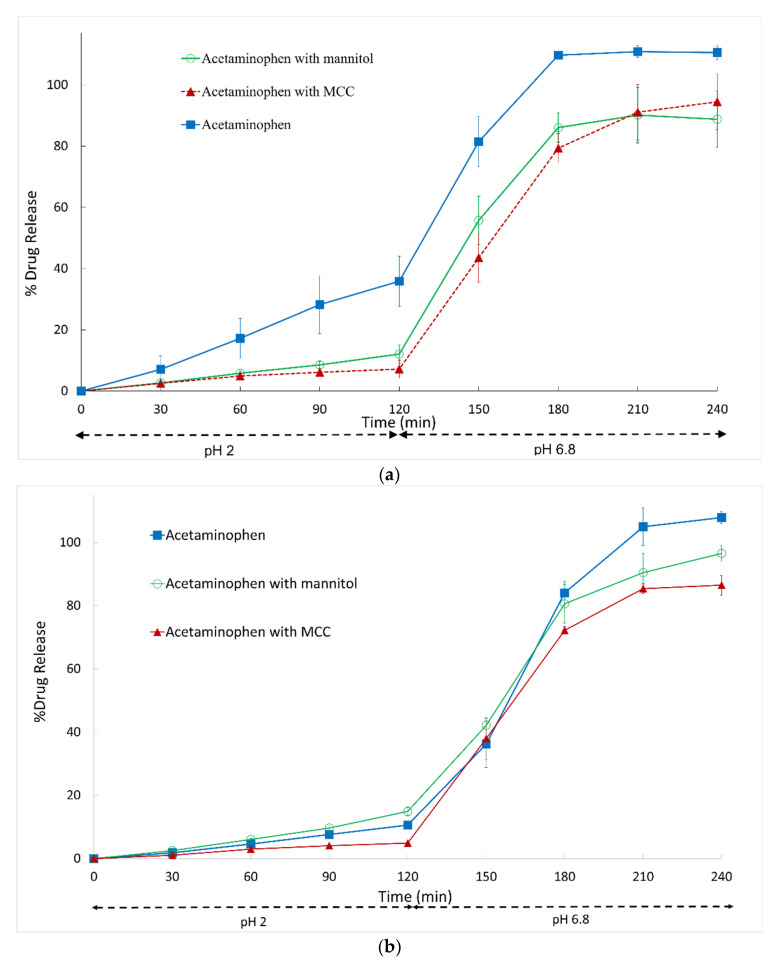
Dissolution profiles of Vcaps^®^ Enteric capsules filled with acetaminophen only or acetaminophen with diluent: (**a**) acetaminophen 20 mg with or without 280 mg diluent; (**b**) acetaminophen 100 mg with or without 200 mg diluent. Data shown as drug release mean % ± SD.

We focused on excipient blend studies for the two model drugs, acetaminophen and atenolol, since acetaminophen is non-ionizable with high solubility and atenolol is the most basic. An increase in capsule-fill load with MCC as a diluent resulted in a reduction in premature gastric release of acetaminophen and atenolol from Vcaps Enteric capsules. Increasing capsule-fill load of 20 mg acetaminophen capsules with MCC to a total fill of 300 mg (7% drug, 93% MCC) significantly reduced premature gastric drug release from 35.9 ± 8.3% to 7.2 ± 2.3% (*p* < 0.01) (Figure 3a). Replacing MCC with the water-soluble diluent, mannitol, also significantly reduced premature drug release to 12.1 ± 2.9% (*p* < 0.01) (Figure 3a). A similar extent of premature gastric release of acetaminophen was observed with the addition of either water-soluble or -insoluble diluent (*p* > 0.05). The results demonstrate that increasing the fill load with a diluent can improve capsule integrity by allowing the full body to create pressure on the cap. With low fill loads, however, the cap and body buckle, leading to capsule deformation and premature drug release.

The effect of drug-to-excipient ratio was also explored and capsule-fill load was altered to contain 33% drug (100 mg acetaminophen) and 67% diluent (200 mg MCC or mannitol). Premature drug release was observed to decrease from 10.6% in the absence of diluents to 4.9% with MCC as a diluent (*p* < 0.001). Interestingly, however, the addition of mannitol as a diluent increased premature drug release from 10.6% to 14.9% (*p* < 0.05) (Figure 3b). At the higher drug-to-excipient ratio, mannitol was, therefore, not effective at retarding premature drug release. This may be explained by the presence of more drug at the 100 mg drug load, and highly water-soluble mannitol enables faster dissolution of the capsule fill. MCC, however, is insoluble and, therefore, delays dissolution of the capsule fill. Diluent type was not observed to affect drug release in the intestinal phase.

**Figure 4:**
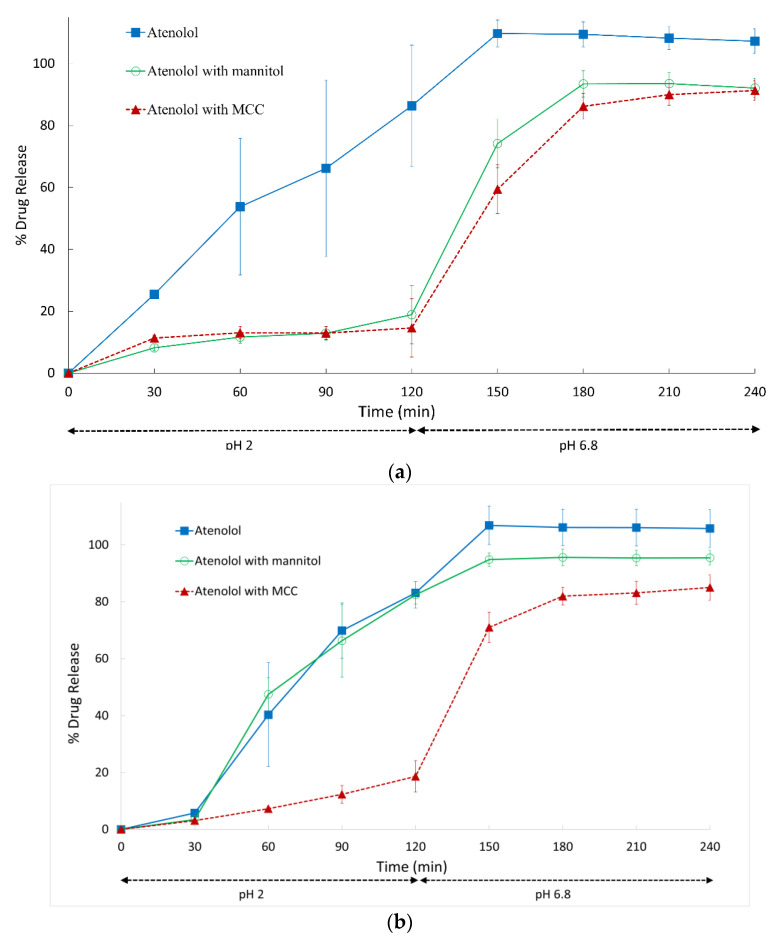
Dissolution profiles of Vcaps^®^ Enteric capsules filled with atenolol only or atenolol with diluent: (**a**) 20 mg atenolol or 20 mg atenolol with 280 mg diluent; (**b**) 100 mg atenolol or 100 mg with 200 mg diluent. Data shown as drug release mean % ± SD.

Similar trends with influence of diluent on premature gastric release of atenolol were also observed. MCC and mannitol significantly reduced premature gastric release at the 7% drug load with 93% diluent. Drug release was reduced from 86.3 ± 19.7% in the absence of diluents to 14.6 % ± 1.4 in the presence of MCC (*p* < 0.05) and to 18.9 % ± 9.4 in the presence of mannitol (*p* < 0.05) (Figure 4a). An increase in drug-to-diluent ratio (33% atenolol, 67% diluent) resulted in a reduction in atenolol premature release from 83.1% ± 4.0 in the absence of diluents to 18.7 ± 5.5% with MCC (*p* < 1 × 10^−4^) (Figure 4b). Addition of mannitol, however, did not significantly change the extent of premature drug release, which remained high at 82.5 ± 4.7% (*p* > 0.05) (Figure 4b). pH*_d_* of atenolol was not found to change in the presence of diluents at the different drug/diluent ratios, remaining in a range of pH 10.3 to 10.5 (Table 2). At the higher drug-to-excipient ratio, mannitol was not effective at retarding premature drug release, as it may lead to faster dissolution of the capsule fill due to its high aqueous solubility.

### 3.3. Effect of Hypochlorhydric, Simulated Fluids on Premature Gastric Drug Release

**Figure 5:**
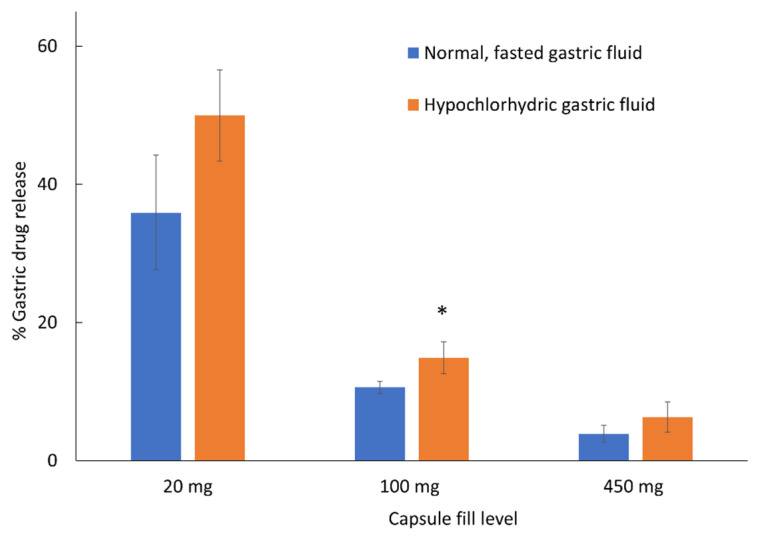
Acetaminophen release from Vcaps Enteric capsules with different fill levels after two-hour exposure to normal, fasted or hypochlorhydric, simulated gastric fluids. * Indicates significantly higher (*p* < 0.05) premature gastric release under hypochlorhydic, simulated fluids compared to normal, fasted simulated gastric fluids. Data shown as drug release mean % ± SD.

**Figure 6:**
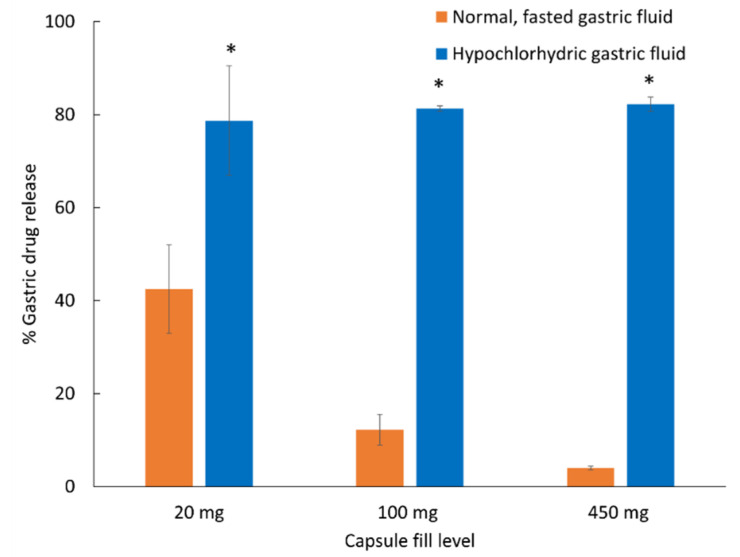
Trimethoprim release from Vcaps Enteric capsules with different fill levels after two-hour exposure to normal, fasted or hypochlorhydric, simulated gastric fluids. * Indicates significantly higher (*p* < 0.05) premature gastric release under hypochlorhydic, simulated fluids compared to normal, fasted simulated gastric fluids. Data shown as drug release mean % ± SD.

Acetaminophen premature gastric release from Vcaps Enteric capsules increased upon two-hour exposure to hypochlorhydric, simulating gastric fluids compared to normal, fasted conditions (Figure 5). However, this increase was only significant at the 100 mg drug-fill level (*p* < 0.05). Trimethoprim premature gastric release under hypochlorhydric conditions significantly increased to ~80% for all the investigated drug loads (*p* < 0.05) (Figure 6). Despite the lower solubility of trimethoprim at pH 4.5 compared to pH 1.2 (0.47 and 3.3 mg/mL, respectively) (Table 1), an increase in gastric drug release may be attributable to the higher pH*_d_*, rising from pH 6.3 ± 0.07 under normal, fasted gastric conditions to pH 8.1 ± 0.18 under hypochlorhydric conditions. pH*_d_*, thus far, exceeds the dissolution pH threshold of the enteric polymer and causes premature dissolution of the enteric capsule.

### 3.4. Implications of Findings

It is imperative to perform systematic in vitro screening of capsule fills when formulating with enteric capsules. In vitro dissolution studies of Vcaps Enteric capsules filled with bisacodyl, budesonide or dimethyl fumarate were conducted and release profiles compared to their enteric-coated, licensed counterparts [23]. Vcaps Enteric capsules filled with the aforementioned drugs were found to be in compliance with pharmacopeial dissolution specifications for enteric formulations [23]. This supports our findings that drug physicochemical properties and capsule-fill level determine the extent of premature gastric release and capsule integrity in the acid phase. Bisacodyl and budesonide are both non-ionizable drugs that are practically insoluble in water. Dimethyl fumarate is highly soluble in water; however, capsule-fill level was high, which may contribute to preservation of capsule integrity in the acid phase. We recognize that the drug doses used in our study may not always reflect the doses used in the clinic. However, the studies conducted here are proof-of-concept studies to alert researchers to necessary investigations with enteric capsules.

Diluents can affect release from immediate-release formulations and, consequently, drug plasma levels. Substituting calcium sulfate with lactose in phenytoin immediate-release capsules was suggested to be responsible for anticonvulsant intoxication in epilepsy patients [24]. Our study shows that the type of diluent and drug-to-diluent ratio can also affect the extent of premature gastric release from enteric capsules.

It is noteworthy that our dissolution study simulated fasted, gastric conditions. Under fed conditions, however, gastric pH will be elevated and gastric-emptying times will be prolonged [25,26], which may further compromise capsule integrity. Physiological variability in gastric transit times has been found to relate to variability in drug pharmacokinetics from gastroresistant dosage forms [27] and in special patient populations [28]. Furthermore, time to disintegration of enteric-coated tablets after gastric emptying was found to correlate with gastric residence times [29]. We also demonstrated that hypochlorhydric-simulated gastric fluids may exacerbate premature gastric drug release from enteric capsules. It is, therefore, imperative to undertake dissolution studies of enteric capsules under conditions simulating both fasted and fed states as well as different patient populations.

## 4. Conclusions

Premature gastric drug release was observed from Vcaps Enteric capsules upon in vitro dissolution testing. The extent of premature drug release is dependent on drug solubility, ionization, capsule-fill level as well as type and ratio of diluent. Premature drug release may be explained by hydration of capsule shell and ingress of aqueous media into capsule fill, causing the drug to diffuse out of the hydrated capsule shell and at the cap/body junction. For weakly basic drugs, ingress of acid media causes the diffusion layer pH to exceed dissolution pH threshold of the enteric polymers, thus, causing premature dissolution of the capsule shell. Hypochlorhydic, simulated gastric fluids may also increase the extent of premature gastric, the significance of which depends on drug physicochemical properties and capsule-fill level. While capsules that are intrinsically enteric offer several advantages, systematic in vitro screening of capsule fills needs to be conducted to ensure that drug product performance is not compromised.

## Figures and Tables

**Table 1:** Physicochemical properties of model drugs.

Drug	Chemical Structure	Ionization	pKa	Solubility (mg/mL)			Dose/ Solubility Ratio (mL) ^f^
				pH 2	pH 4.5	pH 6.8	
Acetaminophen	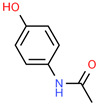	Practically non-ionizable	9.4 ^a,^*	14 ^b^	14 ^b^	14 ^b^	7.1
Trimethoprim	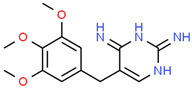	Weak base	7.12 ^a^	3.2 ^c^	0.3 ^c^	-	31.3
Atenolol	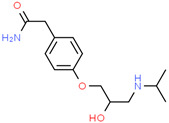	Weak base	9.6 ^a^	20.4 ^c^	-	-	4.9
Ketoprofen	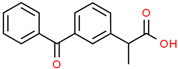	Weak acid	4.39 ^d^	0.21 ^d^	0.49 ^e^	40.8 ^e^	476.2

* Practically non-ionizable over physiological pH range. ^a^ cited from Ref. [16]. ^b^ cited from Ref. [14] (water solubility). ^c^ experimentally determined in house. ^d^ cited from Ref. [18]. ^e^ cited from Ref. [19]. ^f^ dose/solubility ratio for 100 mg drug load in pH 2 acidic media.

**Table 2:** Diffusion layer pH (pH*_d_*) of drug or drug/diluent mixtures at different ratios (data presented as mean ± SD.

Powder Blend	pH*_d_*
Acetaminophen only	2.3 ± 0.02
Acetaminophen with microcrystalline cellulose	
20/280 mg	2.2 ± 0.03
100/200 mg	2.0 ± 0.04
Acetaminophen with mannitol	
20/280 mg	2.0 ± 0.03
100/200 mg	2.0 ± 0.02
Atenolol only	10.3 ± 0.05
Atenolol with microcrystalline cellulose	
20/280 mg	10.3 ± 0.04
100/200 mg	10.6 ± 0.01
Atenolol with mannitol	
20/280 mg	10.3 ± 0.03
100/200 mg	10.5 ± 0.04
Trimethoprim only	6.3 ± 0.07
Ketoprofen only	2.0 ± 0.02

## Data Availability

The data presented in this study are available upon request from the corresponding author.
